# MEC-Enabled Hierarchical Federated Learning for Resource-Aware Device Selection in IIoT

**DOI:** 10.3390/s26041380

**Published:** 2026-02-22

**Authors:** Hu Tao, Duan Li, Bin Qiu, Shihua Liang

**Affiliations:** 1 School of Computer Science and Engineering, Guilin University of Technology, Guilin 541004, China; 18276677135@163.com (H.T.); 15340669724@163.com (D.L.);; 2 Guangxi Communications Investment Technology Co., Ltd., Nanning 530000, China

**Keywords:** industrial internet of things (IIoT), hierarchical federated learning (HFL), dynamics, device selection, resource allocation

## Abstract

Hierarchical federated learning (HFL) combined with the Mobile Edge Computing (MEC) paradigm has attracted extensive research interest in the Industrial Internet of Things (IIoT) due to its ability to deploy computational resources near edge devices and effectively reduce communication overhead. However, in real-world applications, the dynamic participation of edge devices and their diverse training objectives can lead to instability in model convergence, affecting overall system performance. To address this challenge, this paper proposes a device selection strategy based on task completion probability to determine participating devices dynamically in each training round. Furthermore, to balance system resource consumption and model performance, we formulate an optimization objective to minimize the loss function under resource constraints. By leveraging theoretical analysis, we reformulate the objective as a loss upper bound minimization problem related to resource allocation, which is subsequently decomposed into multiple subproblems for iterative solving. Simulation results demonstrate that the proposed method achieves superior resource efficiency and training stability. Compared to the state-of-the-art HFL method, DSRA-HFL reduces the average training delay by approximately 18% and energy consumption by 22% under dynamic conditions, while maintaining a competitive model accuracy. This validates the effectiveness of our joint optimization strategy in practical IIoT scenarios.

## 1. Introduction

With the rapid development of emerging technologies such as 5G communication and Mobile Edge Computing (MEC), the Industrial Internet of Things (IIoT) has become a key enabler of industrial intelligence [1,2]. In typical application scenarios such as industrial manufacturing [3], process control [4] and real-time monitoring [5], IIoT enables industrial data acquisition through massive deployments of smart sensors and vision terminals. Furthermore, data modeling and analysis are performed through edge–cloud collaborative computing to optimize production processes [6,7,8,9,10]. Modern industrial environments, such as automated assembly lines and smart grids, impose stringent requirements on real-time response, operational reliability, and data security [11,12]. Nevertheless, practical IIoT deployments face persistent challenges in model training efficiency and data privacy preservation.

To address data privacy concerns in IIoT, federated learning (FL) has gained traction as a promising solution [9]. It enables devices to collaboratively train global models without uploading local data, thereby enhancing privacy protection [13]. In a typical FL process, IIoT devices first download an initial model from the cloud server. After training local models based on their own data, the updated model parameters are uploaded to the cloud for aggregation [14]. This approach reduces communication overhead by exchanging model parameters rather than raw data. However, frequent parameter exchanges between devices and the cloud can still place a substantial burden on network bandwidth and latency, especially with deep learning models containing massive parameters [15].

To address these challenges, hierarchical federated learning (HFL) has emerged as a promising approach that integrates MEC servers with cloud servers [16,17,18,19,20,21]. In HFL, MEC servers function as intermediate nodes, performing partial aggregation of device models before forwarding the aggregated models to the cloud for global updates [22]. This architecture significantly reduces direct communication between devices and the cloud, thereby decreasing communication latency and resource consumption while preserving overall model performance.

Despite these advantages, real-world IIoT scenarios introduce additional challenges due to the heterogeneity and dynamic nature of edge devices. Devices vary significantly in computing power, communication conditions, energy consumption, and task requirements, making it challenging to optimize resource utilization while ensuring training efficiency. Furthermore, as the number of participating devices increases, efficient device selection and resource allocation under limited resources remain open challenges. Existing HFL approaches often rely on random device selection [23] or fixed, pre-determined device-MEC associations [24]. These strategies overlook the time-varying state information of devices, such as instantaneous computational capability, channel conditions, and task completion probability. For instance, in [23], devices are statically bound to an edge server, which cannot adapt to mobility-induced disconnections. Similarly, the random selection in [24] may frequently include devices with poor channels, leading to ineffective training rounds and resource wastage. Consequently, these strategies fail to fully utilize device resources in dynamic environments, resulting in higher communication overhead and slower model convergence. Efficient device selection and resource allocation play a vital role in improving the effectiveness of HFL, particularly in resource-constrained IIoT environments.

To address these issues, this paper proposes an HFL method, DSRA-HFL, that jointly performs dynamic device selection and resource allocation. In each training round, the proposed approach selects devices with higher task completion probabilities, significantly enhancing overall system performance while reducing training time and communication overhead. Additionally, we formulate an optimization objective to minimize the loss function under resource constraints, balancing resource consumption and model performance. Based on theoretical analysis, we decompose this problem into a series of more manageable subproblems for iterative solving. The main contributions of this paper are as follows:

(1) To address the instability caused by the heterogeneous and time-varying availability of edge devices, we propose a novel HFL method called DSRA-HFL. It employs a dynamic device selection strategy based on real-time task completion probability, which prioritizes devices with higher likelihood of successful participation, thereby enhancing training process stability.

(2) We formulate a joint optimization problem that captures the interplay between device selection and multi-dimensional resource allocation (computing, communication). By theoretically transforming and decomposing this complex problem, we provide a tractable solution that balances global model performance with strict system resource constraints.

(3) Even under a simplified mobility assumption (modeled as varying distances), the evaluation demonstrates that *DSRA-HFL*’s joint optimization framework effectively mitigates performance fluctuations and outperforms benchmarks in terms of training efficiency and resource utilization, validating the necessity of co-designing selection and allocation.

The remainder of this paper is organized as follows: Section 2 reviews the related work on FL from several aspects. Section 3 presents the system model and formulates the problem. The process of solving the original problem using iterative optimization is detailed in Section 4 and demonstrated through some simulation experiments in Section 5. Section 6 summarizes the work of this paper.

## 2. Related Work

In recent years, numerous studies on IIoT systems have focused on the performance of FL, improving communication efficiency, and enhancing resource utilization. To reduce the impact of communication overhead on FL, [25] introduced a novel projection-based local model compression scheme, leveraging the relevance of local model updates to significantly enhance FL communication efficiency. Considering network congestion caused by the heterogeneity of IoT devices and networks, [26] developed a knowledge migration-based FL framework that utilizes knowledge distillation techniques to optimize FL, effectively reducing the burden on edge networks. In [27], a digital twin model was constructed using data collected from IoT devices in edge networks. An asynchronous updated FL scheme was designed to minimize communication overhead, while a convolutional neural network model was employed to decompose the communication cost optimization problem, achieving an optimal allocation of communication resources. Similarly, Ref. [28] proposed a personalized FL scheme that fine-tunes the local learning rate and iteratively solves the communication resource allocation problem. This approach integrates heterogeneous data and limited resources to enhance system adaptability.

Traditional FL relies on a centralized server for model aggregation. However, with the rapid increase of IoT devices, network congestion and inefficient training have become more severe, making it difficult to meet user demands. To address this issue, HFL architecture incorporating MEC technique has attracted significant attention in recent years [16,17,18,19]. In [16], a joint optimization problem was formulated for computational and communication resource allocation as well as edge association, aiming to minimize global cost. Their approach first applies an optimal policy for the resource allocation subproblem within individual MEC servers, followed by an iterative cost-reduction strategy to associate IoT devices with MEC servers efficiently. To further alleviate edge devices’ dependence on the central server, [17] introduced a two-tier resource allocation and incentive mechanism, where the selection of edge devices is modeled as an evolutionary game. Additionally, a deep learning-based auction mechanism was employed to determine the head device in each set to maximize the seller’s revenue. In [18], a hierarchical game framework was presented that captures the dynamic nature of HFL by integrating resource allocation, device scheduling, and incentive mechanisms. Meanwhile, [19] designed a joint device scheduling and communication resource allocation scheme to balance gradient dispersion and resource consumption, achieving a trade-off between training efficiency and network resource utilization.

The aforementioned studies indicate that selecting an appropriate subset of devices for FL can enhance training efficiency and reduce communication latency. However, they do not fully consider the dynamic nature of device participation. To address this limitation, some studies have explored potential solutions. In FL-assisted telematics, vehicles frequently move out of MEC server coverage areas, leading to a decline in global model accuracy. In [20], an asynchronous FL and deep reinforcement learning-based approach was proposed to achieve optimal caching for in-vehicle application content prediction. Similarly, in order to improve mobile client awareness in IIoT environments, [21] developed an incentive-based FL framework and designed a knowledge distillation algorithm to mitigate the issue of non-IID data distribution.

Despite these advancements, research on device dynamics in HFL remains limited. In response, this paper investigates a dynamic IIoT scenario where smart devices, such as mobile phones and vehicles, are in motion. This mobility requires the set of selected devices to be updated dynamically in each training round, and leads to fluctuating wireless channel conditions, which poses significant challenges for communication resource allocating. Taking these considerations into account, this study optimizes FL performance through joint device selection and resource allocation.

## 3. System Model

### 3.1. HFL Model

**Figure 1:**
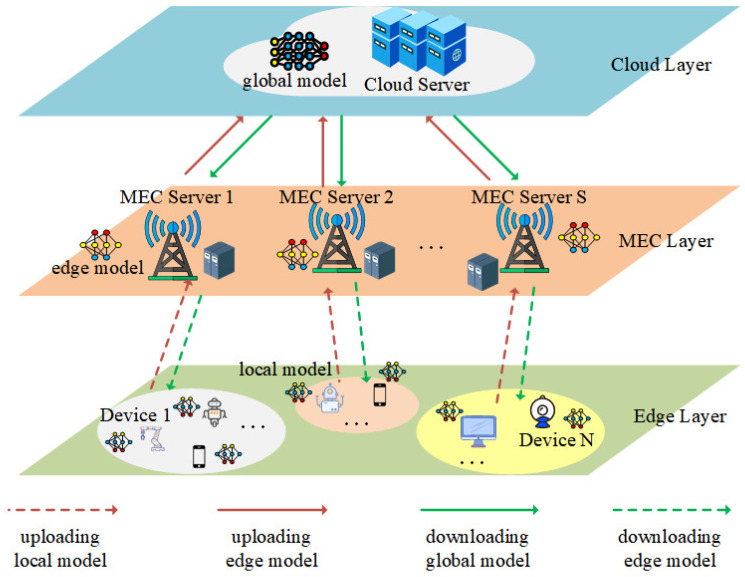
HFL system model for IIoT scenario.

Figure 1 illustrates the three-layer architecture of the IIoT scenario considered in this paper, comprising the cloud layer, MEC layer, and edge layer. The cloud layer contains a set of high-performance servers. The MEC layer contains *M* servers, denoted by the set $M=\left\{1,\ldots ,m,\ldots ,M\right\}$. Each MEC server is equipped with a signaling base station exchanging the model with the cloud layer and the edge layer through wireless communication. The edge layer consists of *N* IIoT devices such as cell phones, computers, robotic arms, cameras, etc., represented by the set $N=\left\{1,2,\ldots ,N\right\}$. $D_{n}={\left\{\left(x_{i},y_{i}\right)\right\}}_{i=1}^{D_{n}}$ denotes the dataset on the device *n*, $x_{i}$ represents the *i*-th input sample, and $y_{i}$ represents the corresponding output label. $D_{n}$ denotes the size of the data; the total amount of data from all devices is $D=\sum_{n=1}^{N}D_{n}$. In addition, the set of devices within the communication range of the MEC server *m* is defined as $N_{m}$, whose amount is $\left|N_{m}\right|$, and the diameter of the server *m* coverage is $\Phi_{m}$.

The typical process of HFL is as follows: in each round, the cloud layer first broadcasts an initial global model to the MEC layer. Devices in the edge layer obtain this model from the MEC layer and train it based on the local dataset. The updated local model is then uploaded to the MEC layer, where it is aggregated into an edge model. the MEC servers in turn upload their respective edge models to the cloud layer for aggregation to generate a new global model and redistribute it. The above process is repeated for *T* rounds until the global model reaches convergence and the training task is completed.

IIoT devices first update the local model based on the local dataset. For device *n*, the corresponding loss function on its local dataset is denoted as

(1) $$F_{n}\left(\omega_{n}\right)=\frac{\sum_{i=1}^{D_{n}}f\left(x_{i},y_{i},\omega_{n}\right)}{D_{n}},$$

where $f\left(x_{i},y_{i},\omega_{n}\right)$ denotes the loss function, which depends on the type of training task, and $\omega_{n}$ denotes the model parameters of device *n*. We simplify the analysis by assuming that the loss function is smooth and strongly convex, which is denoted as [29].

**Assumption** **1.** *$F_{n}\left(\cdot \right)$ is L-smooth, $\forall \omega_{n,t},\omega_{n,t-1}\in R^{d}$; then,*

(2) $$\begin{array}{c} F_{n}\left(\omega_{n,t}\right)-F_{n}\left(\omega_{n,t-1}\right)\leq \langle \nabla F_{n}\left(\omega_{n,t-1}\right),\omega_{n,t}-\omega_{n,t-1}\rangle \\ +\frac{L}{2}{∥\omega_{n,t}-\omega_{n,t-1}∥}^{2}. \end{array}$$

**Assumption** **2.** *$F_{n}\left(\cdot \right)$ is μ-strongly convex, $\forall \omega_{n,t},\omega_{n,t-1}\in R^{d}$; then,*

(3) $$\begin{array}{c} F_{n}\left(\omega_{n,t}\right)-F_{n}\left(\omega_{n,t-1}\right)\geq \langle \nabla F_{n}\left(\omega_{n,t-1}\right),\omega_{n,t}-\omega_{n,t-1}\rangle \\ +\frac{\mu }{2}{∥\omega_{n,t}-\omega_{n,t-1}∥}^{2}. \end{array}$$

The process of updating the local model by device *n* in the *t*-th round is given by

(4) $$\omega_{n,t}=\omega_{n,t-1}-\eta_{t}\cdot \nabla F_{n}\left(\omega_{n,t-1}\right),$$

where $\eta_{t}$ denotes the learning rate and $\nabla F_{n}\left(\omega_{n,t-1}\right)$ denotes the gradient of the loss function. Then, device *n* uploads the local model parameters to the MEC server *m* and generates the edge model based on (5)

(5) $$\omega_{m,t}=\frac{\sum_{n=1}^{N}a_{n,t}\cdot D_{n}\cdot \omega_{n,t}}{\left|D_{K_{m,t}}\right|},$$

where the decision variable $a_{n,t}\in \left\{0,1\right\}$ indicates whether device *n* participates in the *t*-th edge aggregation of MEC server *m*. $\left|D_{K_{m,t}}\right|$ denotes the total data volume of the selected devices under MEC server *m* in round *t*. The set of these devices is denoted as $K_{m,t}=\left\{n\left|a_{n,t}=1,\forall n\in N_{m}\right.\right\}$. Subsequently, the cloud layer receives all the edge models and aggregates them to generate the global model, which is denoted as

(6) $$\omega_{t}=\frac{\sum_{m=1}^{M}\left|D_{K_{m,t}}\right|\cdot \omega_{m,t}}{D}.$$

To focus on the core problem of joint device selection and resource allocation, we adopt two simplifying assumptions: (1) The channel between MEC servers and the cloud is assumed to be stable and of high bandwidth, as they are typically connected via wired or dedicated high-capacity wireless backhaul, making its impact on the hierarchical aggregation latency secondary. (2) Device mobility is abstracted as changes in distance $d_{n,t}$ per round. While real movement is more complex, modeling it as discrete, linear changes per training round (which typically lasts seconds to minutes) is a common and tractable approximation for studying communication resource allocation in FL. These assumptions allow us to derive a solvable optimization problem while capturing the essence of device dynamics.

### 3.2. Device Dynamics Model

In practical IIoT scenarios, the set of available devices for training is dynamic due to factors such as fluctuating wireless channel conditions, varying battery levels, and mobility-induced changes in network topology. To capture the essence of this dynamic participation while maintaining analytical tractability for the resource allocation problem, we model the primary dynamic factor as the time-varying distance $d_{n,t}$ between device *n* and its associated MEC server in round *t*. This distance variation abstracts the effects of mobility or changing environments, directly impacting the channel gain and task completion probability. We acknowledge that more complex mobility patterns and channel models (e.g., fast fading) exist and are reserved for future extension.

### 3.3. Delay Model

The delay generated by the HFL process typically consists of both local computation and parameter transmission. We define the number of local iterations of the IIoT device in each round as $Q=\upsilon \cdot \log\left(1/\theta \right)$, υ is a constant related to the learning task, and θ represents the local model error level [30]. Then the latency incurred by device *n* to complete local training in the *t*-th round is denoted as

(7) $$T_{n,t}^{cmp}=\frac{c_{n}\cdot D_{n}\cdot Q}{f_{n,t}},$$

where $c_{n}$ and $f_{n,t}$ denote the number of CPU cycles required by device *n* to compute a unit of data and its CPU frequency for this round, respectively. Edge aggregation is then performed and the wireless communication considered in this paper is based on orthogonal frequency division multiple access (OFDMA) technology. Considering the limited communication resources, we assume that in each round, the MEC server *m* assigns *K* subcarriers to $\left|K_{m,t}\right|$ devices, $K<\left|K_{m,t}\right|$, and each device is assigned at most one subcarrier. Thus, the transmission rate of the model parameters for device *n* is

(8) $$R_{n,t}^{com}=s_{n,t}\cdot B\cdot \log_{2}\left(1+\frac{p_{n,t}\cdot h_{n,m,t}\cdot d_{n,m,t}^{-\gamma }}{s_{n,t}\cdot B\cdot N_{0}}\right),$$

where *B* denotes the uplink bandwidth, $s_{n,t}=\sum_{k=1}^{K}u_{n,k}\cdot b_{n,k,t}$, and $b_{n,k,t}\in \left(0,1\right)$ denotes the proportion of the total bandwidth that device *n* is assigned subcarrier *k*. We assume orthogonal frequency division multiple access (OFDMA), where each subcarrier is exclusively assigned to one device within a cell. This represents a proportion of the time-frequency resource block, not a power splitting among users sharing the same subcarrier. The decision variable $u_{n,k}=\left\{0,1\right\}$ denotes whether subcarrier *k* is assigned to device *n* and $\sum_{k=1}^{K}u_{n,k}=1$. $p_{n,t}$ and $h_{n,m,t}$ denote the transmit power of the device at at the *t*-th round and the channel gain between it and the MEC server, respectively. $N_{0}$ denotes the noise power spectral density. $d_{n,m,t}$ denotes the distance between the device and the MEC server and γ is the path loss exponent. We use $z_{n}$ to denote the size of data uploaded by the device for the local model parameters; then, the transmission delay caused by the device *n* in the *t*-th round is

(9) $$T_{n,t}^{com}=\frac{z_{n}}{R_{n,t}^{com}}=\frac{z_{n}}{s_{n,t}\cdot B\cdot \log_{2}\left(1+\frac{p_{n,t}\cdot h_{n,m,t}\cdot d_{n,m,t}^{-\gamma }}{s_{n,t}\cdot B\cdot N_{0}}\right)}.$$

In this paper, we consider the synchronous transmission. Then, the delay incurred in this round for the set of devices participating in the edge aggregation at the MEC server *m* is

(10) $$T_{m,t}^{edge}=\underset{n\in K_{m,t}}{max}\left\{T_{n,t}^{cmp}+T_{n,t}^{com}\right\}.$$

Due to the small number of MEC servers and their ability to operate stably, we can assume that the channel states of the MEC layer and cloud layer and the transmission power of the MEC servers remain constant during the training process [31]. Therefore the transmission rate and power of the MEC server for transmitting the edge model parameters are defined as ${\bar{R}}_{m}^{com}$ and ${\bar{p}}_{m}$ respectively. And its transmission delay incurred during this round of global aggregation is

(11) $$T_{m,t}^{cloud}=\frac{z_{m}}{{\bar{R}}_{m}^{com}},$$

where $z_{m}$ is the size of edge model. Notably, in the edge aggregation and global aggregation phases, according to (5) and (6), it can be seen that the MEC server and the cloud server only need to perform the weighting operation once in each round. Therefore, the time of parameter aggregation can be ignored due to their powerful computational power. Meanwhile, when the updated global models are redistributed, since their downlink transmission power is much larger than the uplink transmission power of the IIoT devices, the delay can also be ignored. Thus, the total delay in the *t*-th round can be expressed as

(12) $$T_{t}^{total}=\underset{m}{max}\left\{T_{m,t}^{edge}+T_{m,t}^{cloud}\right\}.$$

### 3.4. Energy Consumption Model

Based on the previous considerations, the energy consumption generated during the training process also involves both local computation and parameter transmission phases. For device *n*, the local training energy consumption in the *t*-th round is denoted as

(13) $$E_{n,t}^{cmp}=c_{n}\cdot Q\cdot D_{n}\cdot {\left(f_{n,t}\right)}^{2}\cdot \delta ,$$

where δ denotes the effective capacitance factor of the device’s computational module. The transmission energy consumption generated by device *n* in this round of edge aggregation can be expressed as

(14) $$\begin{array}{c} E_{n,t}^{com}=p_{n,t}\cdot T_{n,t}^{com}\\ =p_{n,t}\cdot \frac{z_{n}}{s_{n,t}\cdot B\cdot \log_{2}\left(1+\frac{p_{n,t}\cdot h_{n,m,t}\cdot d_{n,m,t}^{-\gamma }}{s_{n,t}\cdot B\cdot N_{0}}\right)} \end{array}.$$

Then, the energy consumption generated by the set of devices participating in edge aggregation at the MEC server *m* is

(15) $$E_{m,t}^{edge}=\sum_{n\in K_{m,t}}\left(E_{n,t}^{cmp}+E_{n,t}^{com}\right).$$

The energy consumption generated by transmitting the edge model of the MEC server is $E_{m,t}^{cloud}={\bar{p}}_{m}\cdot T_{m,t}^{cloud}$. Combined with (15), the total energy consumption generated in this round is expressed as

(16) $$E_{t}^{total}=E_{m,t}^{edge}+E_{m,t}^{cloud}.$$

### 3.5. Problem Formulation

In fact, different IIoT devices usually have different training objectives, e.g., some devices focus on improving model performance while others focus on reducing resource consumption. In order to balance them, this paper aims to minimize the global loss function while guaranteeing delay and energy consumption, which can be represented as

$$\begin{array}{cc} \underset{a_{n,t},s_{n,t}}{min}F\left(\omega ,a_{n,t}\right) & \;\;\;\;\;\;\;\;\;\;\;(17)\\ s.t.\;\;\;\;\;\;\;\;a_{n,t}\in \left\{0,1\right\} & \left(17a\right)\\ \underset{n}{max}\left\{a_{n,t}\cdot \left(T_{n,t}^{cmp}+T_{n,t}^{com}\right)\right\}\leq T_{\max} & \left(17b\right)\\ \sum_{m=1}^{M}\left(E_{m,t}^{edge}+E_{m,t}^{cloud}\right)\leq \tilde{E}, & \left(17c\right) \end{array}$$

where constraint (17a) denotes the decision variables for IIoT devices to participate in edge aggregation. Constraints (17b) and (17c) limit the maximum latency for local training and model transmission of the IIoT device and the energy budget for each round, respectively.

## 4. Solution to the Problem

Unfortunately, the dynamic involvement of the device causes the wireless channel state to be variable during training, leading to uncertain resource consumption. Since the loss function does not have a closed-form expression, the problem in (17) is difficult to solve directly. In this regard, we first formulate a device participation scheme and adjust the aggregation strategy according to the completion probability of the task.

**Figure 2:**
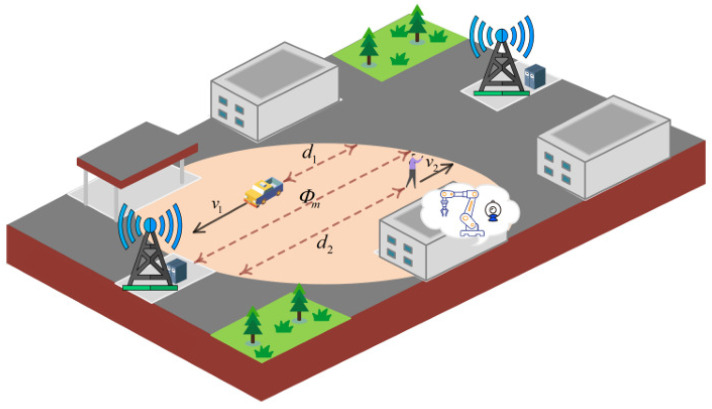
Edge scenario.

As shown in Figure 2, some of the devices (e.g., cell phones and smart vehicles) are in motion and may leave the communication range during the training process. Let the movement speed of device *n* be denoted as $\nu_{n}$, and its distance from the edge of the MEC server’s coverage range at a certain time in the *t*-th round be $d_{n,t}^{edge}$. To simplify the problem, we assume that the device moves at a uniform speed in one direction along the straight line it is in with the MEC server. Based on this, we define the maximum duration for device *n* to successfully complete the training tasks (local training and model transmission) as

(18) $$\tau_{n}=min\left\{\frac{\Phi_{m}-d_{n,t}^{edge}}{\nu_{n}},T_{max}\right\}.$$

Thus the probability that device *n* successfully completes the training task can be expressed as $a_{n,t}^{success}=P\left(T_{n,t}^{cmp}+T_{n,t}^{com}\leq \tau_{n}\right)$. Due to the difficulty in obtaining channel state information (CSI), we assume that the uplink channel obeys Rayleigh fading; thus, $a_{n,t}^{success}$ will be transformed into [32]

(19) $$a_{n,t}^{success}=\left\{\begin{array}{c} 0,\;\;\tau_{n}\leq T_{n,t}^{cmp}\\ \xi_{n,t},\;\;otherwise \end{array}\right..$$

Here, we denote the probability that device *n* successfully completes the training task as $\xi_{n,t}$, and $\xi_{n,t}=exp\left(-\frac{s_{n,t}\cdot B\cdot N_{0}\cdot \left(2^{\frac{z_{n}}{s_{n,t}\cdot B\cdot \left(\tau_{n}-T_{n,t}^{cmp}\right)}}-1\right)}{p_{n,t}\cdot d_{n,m,t}^{-\gamma }}\right)$. In addition, device *n* does not satisfy constraint (17b) and still has $a_{n,t}^{success}=0$, and thus, the constraint (17b) can be removed. Since the number of subcarriers $K<\left|K_{m,t}\right|$ can be assigned by the MEC server in each round, not all devices are guaranteed to be allocated subchannels. It depends on the value of $a_{n,t}^{success}$, even if a device completes local training and model transmission. In this regard, we redefine $a_{n,t}$ as $a_{n,t}=1\left(\xi_{n,t}\in \xi_{K,t}\right)$ by ranking the task completion probabilities of the $\left|K_{m,t}\right|$ devices in descending order and denote the first *K* elements as $\xi_{K,t}$, where $1\left(\cdot \right)$ represents the indicator function. Specifically, when $\xi_{n,t}\in \xi_{K,t}$, $a_{n,t}=1$, and vice versa, $a_{n,t}=0$. This strategy allows for the selection of the “faster” device in each round to fit the limited communication resources, but it also introduces another problem, i.e., the imbalance caused by the difference in the number of rounds in which the devices participate. To mitigate it, we reformulate the edge aggregation process in (5) by including the device’s success probability and resource allocation as additional weights as [33]

(20) $$\omega_{m,t}=\sum_{n=1}^{\left|K_{m,t}\right|}\sum_{k=1}^{K}\rho_{n}\cdot \frac{q_{n,k,t}}{s_{n,t}\cdot \xi_{n,t}}\cdot \omega_{n,t},$$

where $\rho_{n}=\frac{D_{n}}{\left|D_{K_{m,t}}\right|}$, and $q_{n,k,t}=1\left(u_{n,k}=1\wedge R_{n,t}^{com}>\frac{z_{n}}{\tau_{n}-T_{n,t}^{cmp}}\right)$ denotes the joint indicator variable. In order to prove that this aggregation strategy ensures model convergence, we need to make the following assumptions:

**Assumption** **3.** *The variance of the gradient for each device is bounded for a random uniformly sampled portion of data $\zeta_{n,t}$ from the dataset of device n: $E{∥\nabla F_{n}\left(\omega_{n,t},\zeta_{n,t}\right)-\nabla F_{n}\left(\omega_{n,t}\right)∥}^{2}\leq \varsigma_{n}^{2}$.*

**Assumption** **4.** *The expected quadratic moments of the stochastic gradient for all devices are bounded: $E{∥\nabla F_{n}\left(\omega_{n,t},\zeta_{n,t}\right)∥}^{2}\leq G_{n}^{2}$.*

Similarly, the gradient variance and expected quadratic moments of the edge model are denoted as $\varsigma_{m}^{2}$ and $G_{m}^{2}$. When assumptions 1 to 4 hold, compared to the case where all devices are involved, partial device involvement still ensures that the model eventually converges. And after *T* rounds of training, there exists an upper limit of error between the expected value and the minimum value of the global loss function, which can be expressed as [34]

(21) $$E\left[F\left(\omega_{T}\right)\right]-F^{*}\leq \frac{2L}{\lambda +T-1}\cdot \left(\chi_{g}+8Q^{2}G_{g}^{2}+4Q^{2}G_{g}^{2}\beta_{g}\right),$$

where, λ=max8L8Lμμ,Q, $\chi_{g}=\sum_{m=1}^{M}\rho_{m}^{2}\cdot \left(\sum_{n=1}^{\left|K_{m,t}\right|}\rho_{n}^{2}\cdot \varphi_{n}^{2}\right)+6L\cdot \Gamma_{g}+\frac{\mu \cdot \lambda }{2}E{∥\omega_{1}-\omega^{*}∥}^{2}$ and ρm=DKm,tDKm,tDD, $\Gamma_{g}=F^{*}-\sum_{m=1}^{M}\rho_{m}\cdot F_{m}^{*}$ denotes the degree to which the data are not independently and identically distributed at the global level. $G_{g}^{2}=\sum_{m=1}^{M}\rho_{m}^{2}\cdot G_{m}^{2}$ and $\beta_{g}=\sum_{m=1}^{M}\rho_{m}\cdot \beta_{m}=\sum_{m=1}^{M}\sum_{n=1}^{\left|K_{m,t}\right|}\rho_{m}\cdot \rho_{n}\cdot \left(\frac{1}{s_{n,t}\cdot \xi_{n,t}}-1\right)$. The form of the minimization loss function in problem (17) can be transformed to minimize the upper bound on the error by Equation (21). To simplify the notation, we abbreviate $K_{m,t}$ to $H=\left\{1,\ldots ,h,\ldots ,H\right\}$. Since other variables are determined, the original problem can be transformed to

$$\begin{array}{cc} P_{0}:\underset{s_{h,t},f_{h,t},p_{h,t}}{min}\sum_{m=1}^{M}\sum_{h=1}^{H}\rho_{m}\cdot \rho_{h}\cdot \frac{1}{s_{h,t}\cdot \xi_{h,t}} & \;\;\;\;\;\;\;\;\;\;(22)\\ s.t.\;\;\;\;\;\;\;\;\sum_{h=1}^{H}s_{h,t}=1 & \left(22a\right)\\ \sum_{h=1}^{H}\left(E_{h,t}^{cmp}+E_{h,t}^{com}\right)\leq {\tilde{E}}_{t} & \left(22b\right)\\ f_{h}^{\min}\leq f_{h,t}\leq f_{h}^{\max} & \left(22c\right)\\ p_{h}^{\min}\leq p_{h,t}\leq p_{h}^{\max} & \;\;,\;\;(22d) \end{array}$$

where constraint (22a) is the limitation of communication resource allocation, constraint (22b) limits the energy consumption of the device in each round, and constraints (22c) and (22d) are the feasible ranges of the device’s CPU operating frequency and transmission power, respectively. Since $s_{h,t},\;f_{h,t},\;p_{h,t}$ in $P_{0}$ are coupled with each other and cannot be solved directly, we first decouple them into three subproblems: communication resource allocation, CPU frequency control, and transmission power allocation. Then, we solve them iteratively by using alternating optimization.

Firstly, we denote $\xi_{h,t}$ as a form containing three constraint variables

(23) $$\xi_{h,t}=exp\left(-\frac{s_{h,t}\cdot B\cdot N_{0}\cdot \left(2^{\frac{z_{h}\cdot f_{h,t}}{s_{h,t}\cdot B\cdot \left(\tau_{h}\cdot f_{h,t}-c_{h}\cdot D_{h}\cdot Q\right)}}-1\right)}{p_{h,t}\cdot d_{h,m,t}^{-\gamma }}\right)$$

Since $\xi_{h,t}$ in $P_{0}$ is in the denominator position, it can be denoted as ${\tilde{\xi }}_{h,t}$ into the numerator position by removing the negative sign from its exponent part. In this regard, for the given $f_{h,t},\;p_{h,t}$, the communication resource allocation subproblem can be represented as

$$\begin{array}{cc} P_{1}:\underset{s_{h,t}}{min}\sum_{m=1}^{M}\sum_{h=1}^{H}\rho_{m}\cdot \frac{\rho_{h}\cdot {\tilde{\xi }}_{h,t}}{s_{h,t}} & \;\;\;\;\;\;\;\;\;\;(24)\\ \;\;\;\;s.t.\;\;\;\;\;\;\;\;(22a),(22b). & \;\;\;\;\;\;\;\;\;\;(24a) \end{array}$$

Let $\Lambda_{1}=\frac{s_{h,t}\cdot B\cdot N_{0}}{p_{h,t}\cdot d_{h,m,t}^{-\gamma }}$, $\Lambda_{2}=2^{\frac{z_{h}\cdot f_{h,t}}{B\cdot \left(\tau_{h}\cdot f_{h,t}-c_{h}\cdot D_{h}\cdot Q\right)}}$, and the summation part of $P_{1}$ is rewritten as $\frac{\rho_{m}\cdot \rho_{h}\cdot exp\left(\Lambda_{1}\cdot \left(2^{\frac{\Lambda_{2}}{s_{h,t}}}-1\right)\right)}{s_{h,t}}$. It can be found that its second-order derivative with respect to $s_{h,t}$ is greater than 0; therefore, it is a convex problem and we solve it by using the Lagrange multiplier method. The corresponding Lagrange function is

(25) $$L\left(q,\lambda \right)=\sum_{m=1}^{M}\rho_{m}.\left(\begin{array}{c} \sum_{h=1}^{H}\frac{\rho_{h}\cdot {\tilde{\xi }}_{h,t}}{s_{h,t}}\\ +\sum_{h=1}^{H}\alpha_{h}\cdot \left(E_{h,t}^{cmp}+E_{h,t}^{com}-{\tilde{E}}_{t}\right)\\ +\beta \cdot \left(\sum_{h=1}^{H}s_{h,t}-1\right) \end{array}\right)$$

Then the Karush–Kuhn–Tucker (KKT) condition is applied to solve the problem, and the optimal communication resource allocation $s_{h,t}^{*}$ is obtained by calculating the partial derivatives of (25) with respect to $s_{h,t}$. When the variables $s_{h,t}$ and $p_{h,t}$ are determined, the CPU frequency control subproblem can be obtained.

$$\begin{array}{cc} P_{2}:\underset{f_{h,t}}{min}\sum_{m=1}^{M}\sum_{h=1}^{H}\rho_{m}\cdot \frac{\rho_{h}\cdot {\tilde{\xi }}_{h,t}}{s_{h,t}} & \;\;\;\;\;\;\;\;\;\;(26)\\ \;\;\;\;s.t.\;\;\;\;\;\;\;\;(22b),(22c). & \;\;\;\;\;\;\;\;\;\;(26a) \end{array}$$

Obviously, the value of the objective function decreases as $f_{h,t}$ increases. And the left part of constraint (22b) is a monotonically increasing function with respect to $f_{h,t}$; thus, the optimal frequency $f_{h,t}^{*}=max\left\{f_{h}^{\max},{\tilde{f}}_{h,t}\right\}$ can be calculated, where ${\tilde{f}}_{h,t}$ is derived from constraint (22c)

(27) $${\tilde{f}}_{h,t}=\sqrt{\frac{{\tilde{E}}_{t}-\frac{p_{h,t}\cdot z_{h}}{s_{h,t}\cdot B\cdot \log_{2}\left(1+\frac{p_{h,t}\cdot h_{h,m,t}\cdot d_{h,m,t}^{-\gamma }}{s_{h,t}\cdot B\cdot N_{0}}\right)}}{c_{h}\cdot Q\cdot D_{h}\cdot \delta }}.$$

Similarly, the transmission power allocation subproblem can be obtained when given $s_{h,t}$ and $f_{h,t}$.

$$\begin{array}{cc} P_{3}:\underset{p_{h,t}}{min}\sum_{m=1}^{M}\sum_{h=1}^{H}\rho_{m}\cdot \frac{\rho_{h}\cdot {\tilde{\xi }}_{h,t}}{s_{h,t}} & \;\;\;\;\;\;\;\;\;\;(28)\\ \;\;\;\;s.t.\;\;\;\;\;\;\;\;(22b),(22d). & \;\;\;\;\;\;\;\;\;\;(28a) \end{array}$$

Due to the non-convexity of constraint (22b), $P_{3}$ is a univariate non-convex optimization problem, for which we solve using the successive convex approximation (SCA) algorithm. First, we define the transmission power of the device in the *j*-th iteration to be $p_{h,t}^{j}$, and $V\left(p_{h,t}^{j}\right)$ denotes the value of $E_{h,t}$, where $E_{h,t}=E_{h,t}^{cmp}+E_{h,t}^{com}$. We approximate the original function by the first-order Taylor expansion of $V\left(p_{h,t}\right)$ at $p_{h,t}^{j}$, which can be expressed as

(29) $$\hat{V}\left(p_{h,t}^{j},p_{h,t}\right)=V\left(p_{h,t}^{j}\right)+V^{\prime }\left(p_{h,t}^{j}\right)\cdot \left(p_{h,t}-p_{h,t}^{j}\right),$$

where $V^{\prime }\left(p_{h,t}^{j}\right)$ represents the first-order derivative of $V\left(p_{h,t}^{j}\right)$ at $p_{h,t}^{*}$. In each iteration, replacing $V\left(p_{h,t}\right)$ with an approximation of (29) ultimately obtains $p_{h,t}^{*}$. Although it is unable to obtain closed solutions for $s_{h,t}^{*}$ and $p_{h,t}^{*}$, we can still perform alternating optimization using block coordinate descent (BCD). First, we define the initial values of the three variables (e.g., the average values), and then fix two variables to optimize the other variable in each iteration until the difference between a certain iteration and the previous round is less than threshold $\psi_{1},\psi_{2},\psi_{3}$, which is finally satisfied in *I* iterations.

The alternating optimization among the three subproblems continues until the changes in the objective function value between consecutive iterations are below a threshold $\epsilon \;=\;10^{-4}$, ensuring convergence to a stationary point. In summary, in the uplink communication phase, the MEC server first selects suitable IIoT devices in each round based on their status and communication resources, and then the device uploads the updated local model, distance, and other parameters such as data size together. In the downlink communication phase, the MEC server sends $s_{h,t}^{*},\;f_{h,t}^{*},\;p_{h,t}^{*}$ along with the updated global model down to the IIoT device. And the device carries out the next round of training based on these parameter settings, and the detailed flow is shown in Algorithm 1.
**Algorithm 1:** An HFL Algorithm for joint device selection and resource allocation (DSRA-HFL).
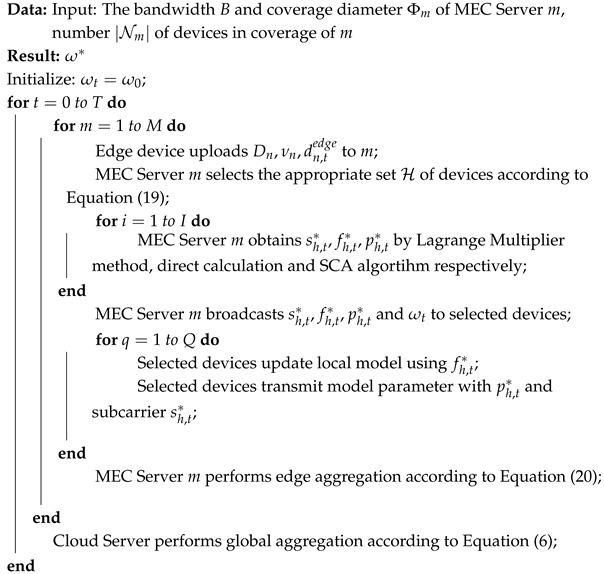


The computational complexity of the above algorithm mainly depends on the number of rounds of FL training, the number of MEC servers, and the number of iterations to solve the three optimal variables. The computational complexity of subproblem 1 using Lagrange multiplier method is $O\left(H^{3.5}\cdot \log\left(1/\psi \right)\right)$. Subproblem 2 can be obtained directly by calculating $f_{h,t}^{*}$; therefore, its computational complexity is ignored. The computational complexity of subproblem 3 using the SCA algorithm is $O\left(H^{3}\cdot J_{SCA}\right)$, where $J_{SCA}$ is the number of iterations of the algorithm. Therefore, the complexity of executing a single run of the BCD algorithm is $O\left(I\cdot \left(H^{3.5}\cdot \log\left(1/\psi \right)+H^{3}\cdot J_{SCA}\right)\right)$. In each round, all MEC servers need to execute the BCD algorithm; therefore, the total complexity of going through *T* rounds is $O\left(T\cdot M\cdot I\cdot \left(H^{3.5}\cdot \log\left(1/\psi \right)+H^{3}\cdot J_{SCA}\right)\right)$. In contrast, the standard HFL baseline [35] that performs fixed resource allocation and device association primarily involves simple averaging operations, with a complexity of approximately O(T·M·H). The increased complexity of *DSRA-HFL* is the necessary cost for performing per-round optimization, which, as shown in Section 5, yields significant gains in resource efficiency. The polynomial complexity in *H* indicates that our method remains scalable and tractable for the considered edge network sizes.

## 5. Numerical Simulation

To verify and assess the performance of the *DSRA-HFL* algorithm, we designed different simulation experiments in the Python 3.10 and TensorFlow 2.0 environments. Additional simulation settings are provided in Table 1. In the simulation experiments, the considered IIoT scenario consists of one central server and three MEC servers, each covering 10 IIoT devices. This scale is chosen to establish a proof of concept for the proposed optimization framework under controlled dynamics. We acknowledge that real-world IIoT deployments may involve hundreds to thousands of devices. The computational complexity analysis in Section 4 indicates that our algorithm scales polynomially with the number of devices, and its efficacy in larger-scale scenarios is an important direction for future work, potentially leveraging clustering techniques [36]. To evaluate the model performance of *DSRA-HFL*, we perform IID and non-IID settings on the MNIST dataset to verify the convergence, respectively. Specifically, 10 handwritten images from the MNIST dataset are included on each IIoT device in the IID setup, while each IIoT device includes any 5 of them in the non-IID setup. In addition, we compare several state-of-the-art baseline methods with *DSRA-HFL*, which are denoted as

*FedAvg* [37]: The central server randomly selects a fixed number of devices to participate in each training round. Communication and computation resources (bandwidth and power) are allocated equally among all selected devices.

*JOFL* [38]: The central server also employs random device selection. It then jointly optimizes the number of local iterations, transmit power, and bandwidth allocation to minimize total system delay and energy consumption for that randomly selected set.

*HFL* [35]: This hierarchical approach statically associates each device with the nearest MEC server at the beginning. Within each round, the MEC server includes all its associated devices for training and jointly optimizes computational resources and bandwidth allocation among them to reduce global communication cost.

*DSRA-HFL*: The MEC server dynamically selects devices in each round based on their real-time task completion probability. It then jointly optimizes transmit power, CPU frequency, and bandwidth allocation specifically for this selected set.

**Figure 3:**
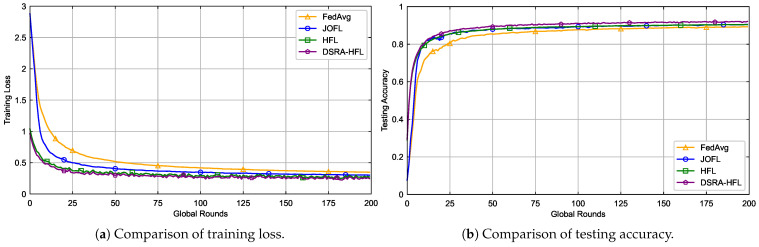
Performance comparison of four algorithms on IID data.

**Figure 4:**
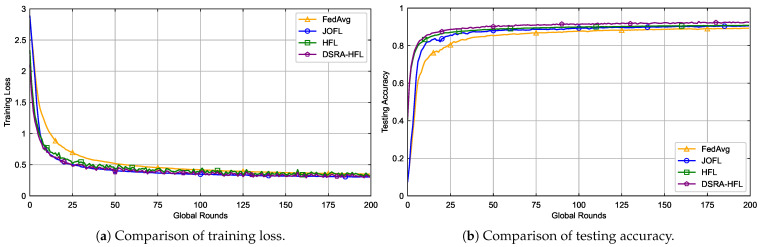
Performance comparison of four algorithms on Non-IID data.

In order to uniform the experimental parameter settings, we first preprocess the MNIST dataset based on the Pytorch framework, allocating 75% of the data for training and 25% for testing. The learning rate is uniformly set to 0.003. Figure 3 and Figure 4 demonstrate the convergence of the four algorithms on the MNIST dataset.

As shown in Figure 3a and Figure 4a, all algorithms eventually converge. The proposed *DSRA-HFL* achieves comparable final accuracy to the *SOTA-HFL* method (within 1% difference), but it attains this level of accuracy with greater stability and fewer oscillations throughout the training process. This is particularly evident in the non-IID setting (Figure 4), where data heterogeneity exacerbates the impact of unstable device participation. *DSRA-HFL*’s dynamic selection proactively filters out devices that are unlikely to meet the delay constraint, creating a more reliable training cohort for each round.

**Figure 5:**
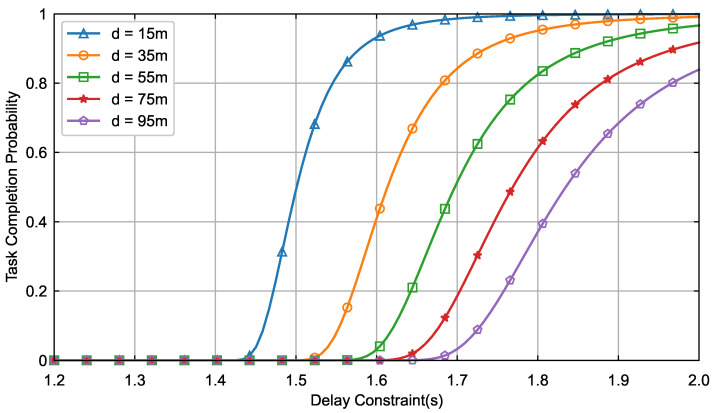
Task completion probability versus delay constraint.

Figure 5 illustrates the quantitative relationship between the task completion probability of terminal devices and the maximum tolerable delay and communication distance. The experimental results show that the task completion probability monotonically increases with the delay constraint, but the long-distance device relies more strongly on the time delay constraint. For example, when d=15 m, it only takes 1.54 s to increase the task completion probability to 0.8, while it takes 1.97 s when d=95 m. Meanwhile, this rise shows a nonlinear trend; for example, the task completion probability grows faster when the delay constraint is increased from 1.6 s to 1.8 s when d=55 m, but slows down when it is increased from 1.8 s to 2.0 s. This indicates that the value of *d* plays a dominant role in the task completion probability. Consequently, the *DSRA-HFL* algorithm tends to prioritize proximity devices while reserving a higher delay budget for the distance device. In addition, when the time delay constraint is less than 1.4 s, the task completion probability is 0 regardless of the value of *d*. This is due to the fact that at this time, the local computation time of the device has already exceeded the delay constraint, and thus, it cannot perform the parameter transmission task.

**Figure 6:**
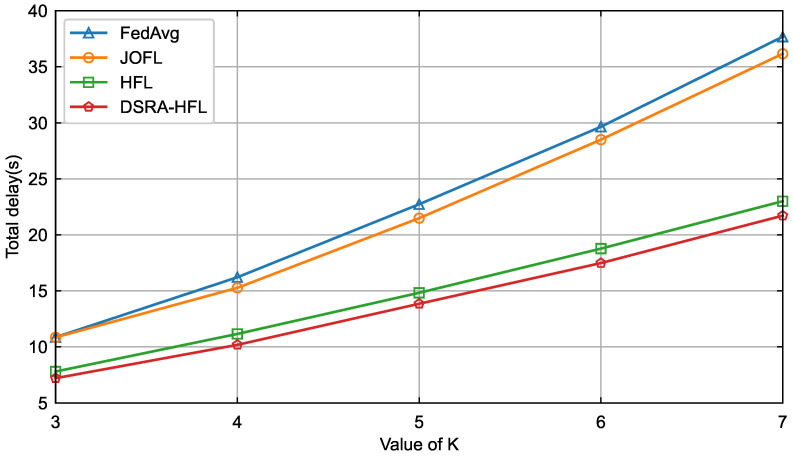
Delay comparison of four algorithms for different values of *K*.

**Figure 7:**
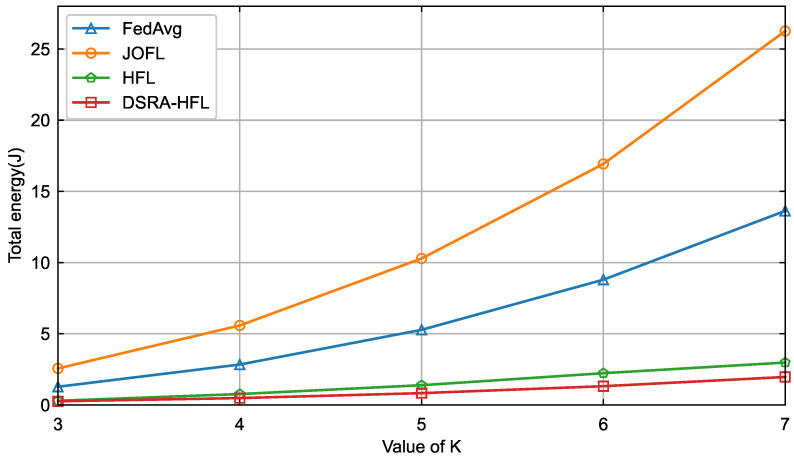
Energy consumption comparison of four algorithms for different values of *K*.

The primary advantage of *DSRA-HFL* is vividly illustrated not in peak accuracy, but in its systemic efficiency. Figure 6 and Figure 7 demonstrate that as the number of candidate devices *K* increases, *DSRA-HFL* curbs the exponential growth in total delay and energy consumption observed in *FedAvg* and *JOFL*. Unlike *HFL*, which employs a fixed association, *DSRA-HFL*’s joint strategy dynamically reallocates resources (bandwidth and power) to the most promising devices in each round. This avoids wasting resources on devices with poor channels or insufficient computational capacity, leading to a superior resource–accuracy trade-off. The near-flat growth curves for *DSRA-HFL* indicate its scalability and suitability for large-scale, resource-constrained IIoT networks.

**Figure 8:**
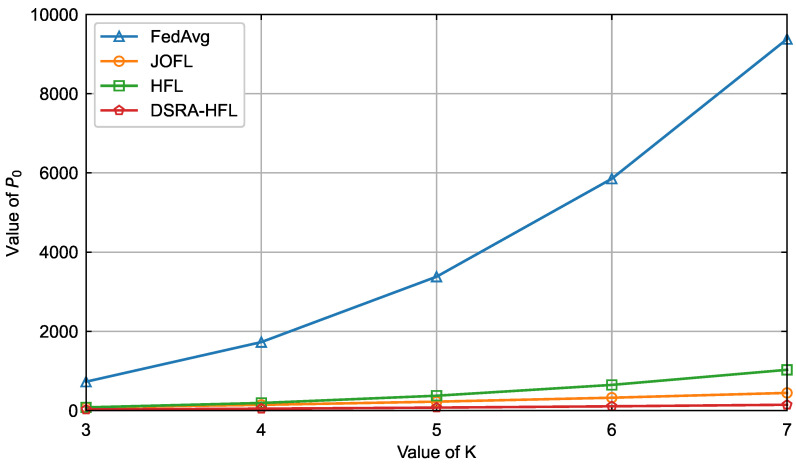
Value of $P_{0}$ versus values of *K*.

Figure 8 shows the value of the optimization objective *P* for different numbers of candidate devices *K*. This is contrary to the naive expectation that more subcarriers (implicitly modeled through finer-grained bandwidth allocations) always lead to lower interference and thus better performance. This occurs because our joint optimization problem balances three competing factors: (1) device selection (favoring reliable devices), (2) bandwidth allocation (combating interference), and (3) energy/delay constraints. Beyond a certain point, adding more candidate devices (or finer resources) introduces more dynamic, low-probability devices into the pool. The *DSRA-HFL* algorithm optimally chooses to ignore these marginal devices rather than over-provision them with resources, leading to a plateau in the loss upper bound. This demonstrates the algorithm’s intelligence in prioritizing stability and resource efficiency over mere resource availability.

## 6. Conclusions

In this paper, a novel HFL method called *DSRA-HFL* is proposed to address the problem of resource dynamic allocation problem caused by device mobility in IIoT. Firstly, the delay model and energy consumption model are constructed for the IIoT scenario. An optimization objective of minimizing the loss function under the constraints of delay and energy consumption is proposed to balance the performance of the model with the efficiency of the system resources. Secondly, a device selection strategy based on the probability of task completion is proposed for the device mobility resulting in the resource dynamic allocation. The original problem is transformed into the problem of minimizing the upper bound of the loss function through theoretical analysis and is decoupled into three subproblems for an iterative solution. Finally, simulation results demonstrate that the proposed method effectively improve the model performance and reduce the system delay and energy consumption compared with existing methods. Future work will investigate more sophisticated client mobility patterns and develop customized training strategies that account for the stationary nature of edge servers and the potential for client disconnections in volatile IoT environments.

## Figures and Tables

**Table 1:** Simulation parameters.

Parameter	Symbol	Value
Local model size	$z_{n}$	1 MB
Edge model size	$z_{m}$	1 MB
Effective capacitance coeff	δ	$10^{-28}$
Path loss exponent	γ	3.5
MEC coverage diameter	$\Phi_{m}$	200 m
System bandwidth	*B*	20 MHZ
Noise power spectral density	$N_{0}$	−174 dBm/HZ

## Data Availability

The original contributions presented in this study are included in the article. Further inquiries can be directed to the corresponding author(s).
